# Identifying parentally perceived barriers for children with celiac disease to participate in elementary school meal programs

**DOI:** 10.1002/jpr3.12141

**Published:** 2024-10-20

**Authors:** Nan Du, Elsa R. Treffeisen, Vanessa Weisbrod, Frances Kelley, Jocelyn Silvester

**Affiliations:** ^1^ Division of Gastroenterology, Hepatology, and Nutrition Boston Children's Hospital Boston Massachusetts USA; ^2^ Division of Immunology Boston Children's Hospital Boston Massachusetts USA; ^3^ Celiac Disease Foundation Woodland Hills California USA

**Keywords:** celiac disease, food insecurity, gluten ingestion, school lunch and breakfast

## Abstract

Several states have recently enacted laws permanently granting all public school students access to free breakfast and lunch. However, children with dietary restrictions, such as celiac disease (CeD), may encounter barriers to participation in these meal programs. We surveyed caregivers of school‐aged children with CeD to study barriers to universal school meals. More than half of the children with CeD did not participate in school meal programs due to concerns about the cafeteria's ability to prepare gluten‐free (GF) meals safely. Moreover, among those who were food insecure and GF food insecure, 50% had never consumed free school lunch and breakfast. Parental perception of nutritional quality, communication regarding GF options, and safety of school kitchens emerged as common obstacles to participation in these programs. Addressing these concerns is paramount to ensuring equitable access to nutritious meals for all students.

## Abstract (highlights)

**What is Known:**

- Gluten‐free (GF) foods are more expensive than their gluten‐containing counterparts.
- Universal school meals remove enrollment barriers and destigmatize receipt of school lunch and breakfast (SLB).

**What is New:**

- More than half of children with celiac disease (CeD) do not participate in school meal programs.
- Half of parents of a child with CeD and food insecurity reported that their child had never participated in school meal programs.
- Parental perception of nutritional quality, communication regarding GF options, and safety of school cafeterias emerged as primary parental perceived obstacles to participation in SLB.

**Abbreviations**
CeD: celiac disease
FI: food insecurity
GF: gluten‐free
IEP: individualized education plan
NSLP: National School Lunch Program
SLB: school lunch and breakfast

## 1 INTRODUCTION

Multiple states (including Massachusetts) have enacted laws to provide funding for universal school meals, thereby enabling all students to receive free school lunch and breakfast (SLB) through the National School Lunch Program (NSLP) and School Breakfast Program.^1^ These laws intend to remove enrollment barriers and destigmatize these programs.^2^ Nevertheless, dietary restrictions, such as a gluten‐free (GF) diet for celiac disease (CeD), may be barriers to participation in these programs. Access to GF meals in school should be documented in a formalized accommodation plan (504 Plan or an Individualized Education Plan [IEP]), which are governed by federal laws, Section 504 of the Rehabilitation Act of 1973 and the Individuals with Disabilities Education Act, respectively. These plans play a crucial role in supporting students with CeD by formalizing GF accommodations, ensuring that school food service staff are informed and prepared to meet these dietary needs. Although all public schools are required to meet the nutritional needs of disabled students (i.e., a GF diet for CeD) at no extra cost to the student,^3^ parents may forgo their child's participation because they perceive that the school does not provide adequate and safe GF options.

At the same time, food insecurity (FI), an economic and social condition of limited or uncertain access to adequate food,^4^ may disproportionally affect people with CeD^5^, ^6^, ^7^ because GF foods are more expensive than their gluten‐containing counterparts.^8^ State and federal food assistance programs often do not account for the increased cost of GF food.^9^ FI can increase the risk of intentional gluten ingestion,^5^, ^6^ which may place individuals at risk of additional autoimmune disorders, intestinal malabsorption, and increased risk of gastrointestinal cancers.^10^ Furthermore, families can screen positive for GF FI even though their household income may be well above the federal poverty line.

Using a survey of caregivers of school‐age children with CeD, we aimed to identify barriers to utilizing universal school meal programs. Our objectives were (1) to characterize the use of free SLB in children with CeD and (2) to understand parental perceptions of these programs among children with CeD. Our hypotheses were that not all children with CeD participated in free SLB and that parents would have negative perceptions of the program.

## 2 METHODS

We used an online platform (REDCap) to survey adult caregivers of children between the ages of 5–11 years who are participants in a community celiac group affiliated with a large tertiary children's hospital. The Celiac Kids Connection group provides support, education and advocacy for families. Caregivers (such as parents) were responsible for decisions about the child's school meal choices. Survey invitations were distributed by email in May 2023 with reminders in June and July 2023. We screened for duplicate emails and cross‐matched to ensure that each household only received one survey. Given that the primary contact for this group is through email communication with undeliverable email addresses removed iteratively, frame error was limited.

The one‐time survey included items related to parental perceived barriers to the acceptance of free school GF meals, including attitudes (trust in the school kitchen to prevent cross contamination), prior experiences (history of gluten exposure, stigma of having “free lunch”) or knowledge (health consequences of persistent gluten ingestion) (Supporting Information). Questions were adapted from prior research on elementary parent perceptions of the NSLP conducted in Virginia and New Jersey.^11^, ^12^ Risk of FI was assessed using the Hunger Vital Sign and a GF adapted version.^6^, ^13^ Demographic and socioeconomic characteristics were self‐reported. The questionnaire was pilot tested. Informed consent was obtained at the beginning of the survey. The Boston Children's Hospital Institutional Review Board approved the study protocol.

### 2.1 Measures

The main outcomes of the study were to assess the participation in free SLB by families with children with CeD in the Massachusetts public school system and to identify perceived barriers to participation in the free SLB. To assess perceptions of SLB, answer options were on a 4‐point Likert scale. Survey questions and response categories can be found in the supplement.

### 2.2 Statistical analysis

Standard descriptive summaries and proportions for categorical variables were used. For groups defined by their responses to attitudinal, prior experiences or knowledge questions on Likert scales, the variable was dichotomized (“very concerned” or “somewhat concerned” vs. “not very concerned” or “not at all concerned”). This increased reliability of data as there were no neutral opinions. All analyses were conducted using R version 3.1.3.^14^

## 3 RESULTS

### 3.1 Sample selection

**Figure 1:**
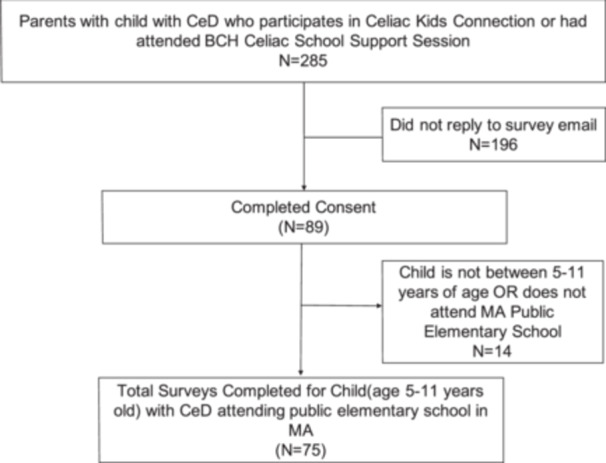
Schematic showing survey questionnaire sample selection. We identified caregivers of children between the ages of 5–11 years who are participants of a community celiac group that is affiliated with one large tertiary children's hospital. Specifically, we used the age reported on their Celiac Kids Connection signup. BCH, Boston Children's Hospital; CeD, celiac disease; MA, Massachusetts.

Of the 285 households who were invited to the survey, 89 completed the consent (Figure 1) of which 75 met the inclusion criteria and submitted a completed survey (26% overall response rate).

### 3.2 Demographics

Most households self‐identified as White (73, 97%), had household income >$100,000 (59, 79%), resided in a mortgaged home (70, 93%), and had high educational attainment (bachelor's degree or higher [71, 95%]). The median child age was 9 years and 62% (47) had been on a GF diet for at least 2 years. Most (84%, 64) had never consumed free school breakfast and 45% (34) had never consumed free school lunch.

### 3.3 FI and use of SLB

The prevalence of positive screen for FI was 13% (10) while 16% (12) screened positive for GF FI. Among those who were food insecure and GF food insecure, 50% (6) had never consumed free SLB. Seventeen percent (13) perceived that they did not have a formalized education plan in place with the school to provide GF meals. Forty‐eight percent (36) felt that their school rarely or never offered GF options for school breakfast and 28% (21) felt that their school rarely or never offered GF options for school lunch.

### 3.4 Communication about GF options

Most families (46, 61%) were somewhat dissatisfied or very dissatisfied with school communication about GF options for SLB of whom 40% (25) perceived that schools do not communicate about GF options at all. The most common ways that GF options were perceived to be communicated were email (27, 39%), school webpage (15, 20%), and phone calls (13, 17%) (Supporting Information: Figure 1a).

### 3.5 Reasons for (not) participating in SLB

The 48 respondents who participated in SLB selected an average 2.7 reasons for participating in SLB. The most common were “child's friends eating school lunch” (82%, 42) and “SLB is convenient” (61%, 31). The other 63 families reported an average of 2.8 reasons for not participating in SLB. The most common reasons were “My family does not like what is being served for SLB” (41, 62%) and “I am not confident that the school cafeteria can make a safe GF meal for my child” (34, 52%). (Supporting Information: Figure 1b,c).

### 3.6 Parental attitudes and trust in school cafeterias

When parents were surveyed about the school's GF food options, 75% (56) felt that there was a “very limited variety” or “not very extensive” GF food options offered at school and 27% (20) felt that the GF food was “not very appealing” or “not at all appealing” for consumption. One out of four (19) parents had “not very much” or “no trust at all” in the school cafeteria to prevent gluten cross‐contact. One in six (12) parents believed their child had an unintentional gluten ingestion while consuming SLB. Approximately 45% (34) were “very concerned” or “somewhat concerned” about gluten cross‐contact at their school's cafeteria. Most parents (61, 82%) considered unintentional gluten ingestion related to SLB to be a “very serious” or a “somewhat serious” health concern.

### 3.7 Parental perception of nutritional quality of SLB

One out of three (25) parents perceived the overall nutritional quality of SLB to be unhealthy or very unhealthy. Parents reported that general GF food was not easy or somewhat difficult to find in the school cafeteria (44, 59%). GF fruit options were perceived as very easy or somewhat easy to find in a school's cafeteria (53, 71%), whereas GF protein‐rich foods and carbohydrates were perceived less easy to find (32, 43% for carbohydrates, and 22, 29% for protein‐rich foods).

## 4 DISCUSSION

SLB programs were introduced over 50 years ago to ensure that students are well‐nourished and ready to learn.^15^ Despite SLB programs offering up to two free meals per day, parents of children with CeD reported that their children were unable to consistently access these meals. Furthermore, 50% of families with a positive screen for FI were not partaking in the free SLB which highlights the underutilization of this resource by families of children with CeD.

As with any study, our design has some limitations. The survey design may have selection bias as the participants who respond to the survey may be different from those who do not. However, our survey response rate of 26% falls within reported ranges in a recent randomized control trial of surveys in the healthcare setting.^16^ Our survey was limited to convenience nonrandom sampling with bias to White and affluent families relative to general population and is specific to one state. Families in higher socioeconomic groups may be more likely to forego free SLB if cost is not an issue; nevertheless, we found that half of families experiencing FI were not participating in SLB, an important finding. Although the Hunger Vital Sign was initially developed for young (<36 months) and lower socioeconomic groups, it has subsequently been validated for use in other populations.^17^ The GF vital sign has not been validated.^6^

Though the stigma around enrolling in a free meal plan may be eliminated when all children are eligible, barriers still exist for students who require a medical diet to maintain their health. Prior research demonstrated that parent's perceptions about school meals may impact student participation in school lunch.^18^ Our survey identified that many parents of children with CeD lack trust and confidence in the school cafeteria. This indicates an unmet need for spaces for children with CeD to develop the skills they require to function in social eating environments where gluten is consumed without developing hypervigilance which increases anxiety and reduces quality of life.^19^

Parents also often perceived poor communication about GF options from the school. The plethora of communication media used may contribute to almost one in four families reporting there were no GF options available. Including GF meal availability and processes on a school website could help families access these programs by learning that they exist. Highlighting naturally GF foods and reporting how they are meeting the USDA nutritional guidelines would allay concerns regarding the nutritional quality of SLB, which are not isolated to parents with children with CeD. Our findings align with previous studies that identified negative perceptions of school meal quality and healthfulness, and parental desire to see more fruits, vegetables, salads and scratch cooked meals.^20^, ^21^ Parental perceived nutritional quality of SLB remains low and seems to suffer especially when children are seeking GF protein‐rich foods. Future qualitative research may help identify what changes (as perceived by parents) are necessary to improve nutritional quality of SLB.

Another barrier that we identified was related to use of accommodations with one in six students with CeD not having a 504 plan or IEP in place. Without a plan in place, school food service staff may be uninformed about the child's need for dietary accommodations. Medical providers, especially gastroenterologists, should ask about 504/IEP plans and inquire about SLB consumption by children with CeD.

Our study is the first to assess parental perceptions of barriers to participation in SLB by their children on a GF diet. As more states move towards universal SLB, additional research and support is needed to ensure that families of children with dietary restrictions are comfortable and feel empowered to access these resources for their child. With growing evidence of FI's impact on health, it is imperative that medical providers also lend their voice to education and advocacy to address these identified barriers.

## CONFLICT OF INTEREST STATEMENT

The authors declare no conflict of interest.

## Supporting information


**Supplemental Digital Content:** Redcap Survey Questionnaire on Parentally Perceived Barriers for Children with Celiac Disease to Participate in Elementary School Meal Programs.

Supporting Information.

**Supplemental Figure 1:** Methods of Communication about SLB and Parental Reasons For and Against Participation in SLB. (a) Distribution of Most Common Methods of Communication Utilized by School Regarding Gluten Meal Options for SLB (b) Distribution of Most Common Reasons Why Families Chose to Participate in SLB. Answers for other answer are included. (c) Distribution of Most Common Reasons Why Families Did Not Chose to Participate in SLB. Answers for other answer are included.
