# Supplementary material for: Identifying parentally perceived barriers for children with celiac disease to participate in elementary school meal programs
Source: JPGN Rep. 2024 Oct 20;5(4):470–4. doi: 10.1002/jpr3.12141 (PMC11600365; doi:10.1002/jpr3.12141)
Supplement: Supplementary file 1 — Supplemental Digital Content: Redcap Survey Questionnaire on Parentally Perceived Barriers for Children with Celiac Disease to Participate in Elementary School Meal Programs. [file JPR3-5-470-s001.pdf]

Parental Perceptions of Access and Acceptance of Gluten-Free School Lunches

1) Do you have a child/ren with celiac disease between the age of 5-11 years old who attends a public elementary school?
    a. Yes → Continue on to next question
    b. No → Route to page saying "We appreciate your interest, but unfortunately your experience does not qualify you for taking part in this particular survey. Thank you for your time. "
2) Including yourself, how many people live in your household?
    a. 1
    b. 2
    c. 3
    d. 4
    e. $\geq$5
3) How many total children (defined as younger than 18 years old) with celiac disease live at home with you?
    a. 1
    b. 2
    c. 3
    d. 4
    e. $\geq$5
    f. None→ Route to page saying "We appreciate your interest, but unfortunately your experience does not qualify you for taking part in this particular survey. Thank you for your time."
4) How many of these children with celiac disease are between the ages of 5-11years old?
    a. 1
    b. 2
    c. 3
    d. 4
    e. $\geq$5

< Programmer Note: Response to Q4 should be equal to OR less than response to Q3>

The following questions are specifically in regards to your child who is between the age of 5-11 years old and attends a public elementary school. If you have more than one child who has celiac disease between the ages of 5-11 years old and attends a public elementary school, please consider only one child when answering the questions. To keep it simple, please choose the child with the most recent birthday.

5) What is your relationship to the child with celiac disease?
    a. Parent (Mother, Father, Stepmother, Stepfather)
    b. Grandparent
    c. Uncle
    d. Aunt
    e. Other
6) How old is the child with celiac disease?
    a. 5
    b. 6
    c. 7
    d. 8
    e. 9
    f. 10
    g. 11

7) How long has this child been on a gluten-free diet for celiac disease?
   a. <6 months
   b. 6-12 months
   c. 1-2 years
   d. >2 years
8) What school district does your child currently attend?

< Programmer Note: Dropdown with schools by town>

9) Does your child currently have a 504 plan with accommodations for gluten free meals and snacks as a student with celiac disease? (A 504 plan is a plan for schools to provide specific accommodations for students with a disability, such as celiac disease)
   a. Yes
   b. No, we have a different type of plan such as Individualized Health Plan or Individualized Educational Plan
   c. No, we do not have any specific type of plan in place
   d. I do not know
10) Does your school offer gluten-free options for school lunch and breakfast?
   a. Always
   b. Often
   c. Sometimes
   d. Rarely
   e. Never
11) How does your school communicate about the gluten-free options at school?
   - ☐ School webpage
   - ☐ Email
   - ☐ Phone Call
   - ☐ Text Message
   - ☐ Paper sent home with child
   - ☐ Other
   - ☐ My school does not communicate about gluten-free options at school

   < Each answer option will have the following frequency scale if it is checked with the question: How often is this approach used? >
      - o Always
      - o Often
      - o Sometimes
      - o Rarely
      - o Never
12) To the best of your knowledge over the last school year, how often does your child eat the free breakfast that is served by the school?
   a. Always
   b. Often
   c. Sometimes
   d. Rarely
   e. Never
13) To the best of your knowledge over the last school year, how often does your child eat the free lunch that is served by the school?
   a. Always
   b. Often
   c. Sometimes
   d. Rarely
   e. Never

14) As far as you know, are each of the following a reason that you choose to have your child eat the school lunch/breakfast. Please select as many reasons why your child/ your family chooses to eat the school lunch/breakfast
< Programmer Note: Randomize order of answers>

a. School Lunch and breakfast(SLB) is nutritious
b. SLB is convenient
c. SLB offers enough food for my child
d. SLB offers variety of choices
e. I save money with SLB
f. My child wants to eat school lunch because his/her friends eat school lunch
g. I want to support SLB because it is an important part of school culture
h. I want to support a program that my tax dollars support
i. Other

< Programmer Note: Please show Q15 only if question 12 answer is Never and question 13 is Never>

15) As far as you know, are each of the following a reason that you choose to pack breakfast/lunch from home. Please select as many reasons why you may choose to send food from home for breakfast/lunch
< Programmer Note: Randomize order of answers>

a. I am not confident that the school cafeteria can make a safe gluten-free meal for my child
b. Child does not like what is being served for school lunch/breakfast
c. Making lunch/breakfast at home means I know what my child is eating
d. I think the food we send from home for breakfast/lunch is healthier
e. Lunch/breakfast from home offers more variety and choice
f. I save money with packed lunch/breakfast from home
g. I save time with packed lunch/breakfast from home
h. My child wants a packed lunch/breakfast from home because his/her friends have a packed lunch/breakfast
i. My child does not like waiting in line for school lunch/breakfast
j. The line is too long for school lunch/breakfast
k. School lunch/breakfast is not organic or sustainable
l. Packed lunch/breakfast from home is more convenient
m. My child ate something with gluten in it at school before
n. Other________________________________

16) As far as you know, has your child ever eaten something by mistake that had gluten in it while having school breakfast or lunch ?

a. Yes
b. No
c. I do not know

17) How often are you planning to have your child partake in school lunches and breakfast in the next school year starting September 2023?

a. Always
b. Often
c. Sometimes
d. Rarely
e. Never

As you respond to the following questions(Q18-Q28), please reflect on the school meals (breakfast and lunch) that were offered at your child's school during the current academic year (September 2022-May 2023).

18) Regardless of whether or not your child eats food provided by his/her school, how would you rate the **nutritional quality of foods** offered at your child's school?
    a. Very unhealthy
    b. Unhealthy
    c. Healthy
    d. Very healthy
    e. Do not know
19) Regardless of whether or not your child eats food provided by his/her school, how easy is it to find **gluten-free food** at your child's school?
    a. Very easy to find
    b. Somewhat easy to find
    c. Not very easy to find
    d. Impossible to find
    e. Do not know
20) Regardless of whether or not your child eats food provided by his/her school, how easy is it to find **gluten-free carbohydrates** at your child's school?
    a. Very easy to find
    b. Somewhat easy to find
    c. Not very easy to find
    d. Impossible to find
    e. Do not know
21) Regardless of whether or not your child eats food provided by his/her school, how easy is it to find **fruit options** at your child's school?
    a. Very easy to find
    b. Somewhat easy to find
    c. Not very easy to find
    d. Impossible to find
    e. Do not know
22) Regardless of whether or not your child eats food provided by his/her school, how easy is it to find **protein-rich food options** at your child's school?
    a. Very easy to find
    b. Somewhat easy to find
    c. Not very easy to find
    d. Impossible to find
    e. Do not know
23) Regardless of whether or not your child eats food provided by his/her school, how easy is it to find **the availability of proteins at school**?
    a. Very available
    b. Somewhat Available
    c. Not very available
    d. Not available at all
    e. Do not know
24) Regardless of whether or not your child eats food provided by his/her school, how would you rate the **variety of gluten-free food options offered in a week** offered at your child's school?
    a. Very extensive
    b. Somewhat extensive
    c. Not very extensive

d. Very limited
e. Do not know

25) Regardless of whether or not your child eats food provided by his/her school, how **visually appealing would you rate the gluten-free food** offered at your child's school?
    a. Very appealing
    b. Somewhat appealing
    c. Not very appealing
    d. Not appealing at all
    e. Do not know
26) Regardless of whether or not your child eats food provided by his/her school, how much do you **trust your school's kitchen to prevent gluten cross contamination**?
    a. A great deal
    b. Somewhat
    c. Not very much
    d. Not at all
    e. Do not know
27) Regardless of whether or not your child eats food provided by his/her school, how **satisfied are you with how your school communicate about gluten-free options available at the school**?
    a. Very satisfied
    b. Somewhat satisfied
    c. Somewhat dissatisfied
    d. Very dissatisfied
    e. Do not know
28) Considering the recent supply chain issues, some gluten free brands that schools used to offer may have been replaced or unavailable. Regardless of whether or not your child eats food provided by his/her school, how satisfied are you with **your school's communication regarding these changes/substitutions to gluten-free options** at school?
    a. Very satisfied
    b. Somewhat satisfied
    c. Somewhat dissatisfied
    d. Very dissatisfied
    e. Do not know

29) If your child were to unintentionally get gluten exposure while consuming lunch/breakfast provided by the school, how serious of a health concern would that be for you?
    a. Very serious
    b. Somewhat serious
    c. Not very serious
    d. Not at all serious
    e. I do not know
30) How concerned are you about your child getting gluten cross contact at school this year?
    a. Very concerned
    b. Somewhat concerned
    c. Not very concerned
    d. Not at all concerned
    e. I do not know
31) How easily accessible are gluten free food options in your local area?
    a. Readily available – I can usually get what I need at just 1 or 2 stores
    b. Available - I regularly shop at 3 or more stores to get what I need
    c. Limited availability – I often go without things I need and/or have to order online
    d. Very poor – I purchase most gluten-free foods online

32) Does your child have any additional dietary restrictions beside gluten free?
    a. Yes
    b. No

For the following statements(Q33-37), please tell us if the statement is often true, sometimes true or never true for {you, your household} in the last 12 months

33) {I/we} have worried about whether our food will run out before we got money to buy more in the last 12 months
    a. Often True
    b. Sometimes True
    c. Never True
34) The food that {I/we} bought just didn't last and {I/we} did not have enough money to get more food
    a. Often True
    b. Sometimes True
    c. Never True
35) {I/we} have worried about whether our **gluten-free food** will run out before we got money to buy more in the last 12 months
    a. Often True
    b. Sometimes True
    c. Never True
36) **The gluten-free food** that {I/we} bought just didn't last and {I/we} did not have enough money to get more **gluten-free food**
    a. Often True
    b. Sometimes True
    c. Never True
37) {I/we} could not afford to eat balanced meals
    a. Often True
    b. Sometimes True
    c. Never True

Definition: Balanced Meal: A balanced meal includes all the types of food that you think should be in a healthy meal. For example, a starch like potatoes or rice, vegetables or fruit and some protein like meat, fish, cheese or eggs.

For the next set of questions, we would like to answer the questions regarding yourself. This information will be kept private and secure.

Demographics

38) What is your home's zipcode ______________________
39) Do you consider yourself
    a. Male
    b. Female
    c. Non-binary
    d. A different gender(Please specify: ____________)
    e. Prefer not to disclose
40) What is your age?
    a. under 18
    b. 18-24
    c. 25-34
    d. 35-44

e. 45-54
f. 55-64
g. 65 or older

41) What is the highest grade or level of school that you have completed?
a. Some High School
b. High School Graduate
c. GED or Equivalent
d. Some College, No Degree
e. Associate’s Degree
f. Bachelor’s Degree
g. Master’s Degree
h. Professor School Degree
i. Doctoral Degree

42) What race or races do you consider yourself to be? Please select one or more
a. American Indian or Alaska Native
b. Asian
c. Black or African American
d. Native Hawaiian or Pacific Islander
e. White
f. Other
g. Do not know

43) Do you consider yourself to be Hispanic, Latino or of Spanish origin?
a. Yes
b. No
c. Do not know

44) How much total combined money did all members of your HOUSEHOLD earn last year?
a. $0-$15,000
b. $15,001-30,000
c. $30,001-45,000
d. $45,001-60,000
e. $60,001-80,000
f. $80,001-100,000
g. more than $100,000
h. prefer not to answer

45) What type of housing do you and your family currently reside in?
a. Homeless/shelter
b. Staying with friends/family
c. Subsidized housing
d. Rental
e. Mortgage/own home
f. Other
