# Supplementary material for: Identifying parentally perceived barriers for children with celiac disease to participate in elementary school meal programs
Source: JPGN Rep. 2024 Oct 20;5(4):470–4. doi: 10.1002/jpr3.12141 (PMC11600365; doi:10.1002/jpr3.12141)
Supplement: Supplementary file 2 — Supporting Information. [file JPR3-5-470-s002.docx]

**Supplemental Additional Data Analysis by Income Level:**

We compared those who were <100,000 household income (8) with those >100,000 income (60). Of note, given the smaller sample size in those with lower household income, the results may be less reliable. Those who were lower income were more likely to have IEP or 504 plan (100%) compared to those with higher income (76%, p=0.07). In terms of SLB participation, those in the higher income had similar participation in the SLB (56%, 34) participate in lunch, 15%, 9, participate in breakfast) compared to those in lower income (46%, 5, participation in lunch, 18% (2) in breakfast)**.** Both groups (higher income vs. lower income) had similar top reasons for participating in SLB: “child’s friends eating school lunch” (55% vs 55%), “SLB is convenient” (47% vs. 27%) and “[they] save money with SLB” (28.3% vs. 45%), although a larger proportion of the higher income group valued convenience while a larger proportion in the lower income group valued saving money. Both groups (higher income and lower income) also had similar reasons for not participating in SLB which included: “Child does not like what is being served for school lunch/breakfast”(64% vs 45%), “I am not confident that the school cafeteria can make a safe GF meal for my child” (47% vs. 64%), and “Making lunch/breakfast at home means I know what my child is eating”(45% vs. 36%).

Parents from the lower income group had higher proportion who were “very concerned” or “somewhat concerned” about their child getting gluten cross contamination in the school’s cafeteria (64%) compared to parents from the higher income group (42%, p=0.18). Parents from both income groups had “not very much” or “no trust at all” in the school cafeteria in preventing gluten cross-contamination (23.3% for higher income and 36.4% for lower income). There was a higher proportion from the lower income group who perceived the SLB nutritional quality of GFF as unhealthy (45%) in comparison to the higher income group (30%, p=0.32). While none of these comparisons were statistically significant, the trends indicate the additional education and efforts toward building trust between parents and school kitchen may have higher impact in populations of lower socioeconomic status.
