# Supplementary material for: Identifying parentally perceived barriers for children with celiac disease to participate in elementary school meal programs
Source: JPGN Rep. 2024 Oct 20;5(4):470–4. doi: 10.1002/jpr3.12141 (PMC11600365; doi:10.1002/jpr3.12141)
Supplement: Supplementary file 3 — Supplemental Figure 1: Methods of Communication about SLB and Parental Reasons For and Against Participation in SLB. (a) Distribution of Most Common Methods of Communication Utilized by School Regarding Gluten Meal Options for SLB (b) Distribution of Most Common Reasons Why Families Chose to Participate in SLB. Answers for other answer are included. (c) Distribution of Most Common Reasons Why Families Did Not Chose to Participate in SLB. Answers for other answer are included. [file JPR3-5-470-s003.docx]

**Supplemental Figure 1**

a)

b)

**Other Parental Response**

“It pushes my child to try new things”

“My child likes the lunch choice that day”

c)

**Other Parental Response:**

“To my knowledge, there is only gluten free pizza on Fridays, which needs to be ordered in advance”

“My daughter also has T1D and the school lunch carb count is very high.”

“My child has multiple food allergies in addition to celiac. That leaves her with the option of only a sun butter sandwich daily, which is too depressing to sign her up for.”

“Sometimes she gets sick of the lack of variety of lunches because it is the same every week.”

“Picky eater with Celiac Disease”

“I supplement the school lunch with a gluten free alternative (e.g. GF waffle on breakfast for lunch day, when everything the school serves is GF except the waffle”

“Daughter with Celiac does not trust eating school lunch.”
